# Serum levels of biomarkers of bone and cartilage destruction and new bone formation in different cohorts of patients with axial spondyloarthritis with and without tumor necrosis factor-alpha blocker treatment

**DOI:** 10.1186/ar2537

**Published:** 2008-10-22

**Authors:** Heiner Appel, Louise Janssen, Joachim Listing, René Heydrich, Martin Rudwaleit, Joachim Sieper

**Affiliations:** 1 Department of Gastroenterology, Infectiology and Rheumatology, Charité Berlin, Campus Benjamin Franklin, Hindenburgdamm 30, 12203 Berlin, Germany; 2 Deutsches Rheumaforschungszentrum Berlin, Schumannstrassse 21/22, 10117 Berlin, Germany

## Abstract

**Introduction:**

Recent data about radiographic progression during treatment with tumor necrosis factor-alpha (TNF-α) blocker agents in patients with ankylosing spondylitis (AS) have prompted an intensive discussion about the link between inflammation/bone destruction and new bone formation and the order of events. Therefore, we analysed parameters of cartilage degradation, neoangiogenesis, and new bone formation in different cohorts of patients with axial spondyloarthritis with and without treatment with TNF-α blocker agents.

**Method:**

TNF-α blocker-naïve AS patients were investigated for serum levels of metalloproteinase-3 (MMP-3) (n = 71), vasoendothelial growth factor (VEGF) (n = 50), and bone-specific alkaline phosphatase (BALP) (n = 71) at baseline and after 1 and 2 years. This was compared with 34 adalimumab-treated patients with axial spondyloarthritis (22 AS and 12 non-radiographic axial spondyloarthritis patients) before and after 36 to 52 weeks of treatment.

**Results:**

There were no significant changes in serum levels of MMP-3 (*P *> 0.05), VEGF (*P *> 0.05), and BALP (*P *> 0.05) in a large cohort of TNF-α blocker-naïve AS patients followed for 2 years. In contrast, adalimumab-treated spondyloarthritis (AS and non-radiographic axial spondyloarthritis) patients had a significant decrease of VEGF (*P *< 0.001) and MMP-3 (*P *= 0.022) after 36 to 52 weeks of therapy. Most interestingly, the level of BALP increased significantly after 36 to 52 weeks of therapy (*P *< 0.001). A decrease in MMP-3 serum levels correlated significantly to an increase of BALP (*r *= -0.398, *P *= 0.02). In the case of VEGF, there was a negative correlation without significance (*r *= -0.214, *P *> 0.05).

**Conclusions:**

Rising levels of BALP and the negative correlation between MMP-3 and BALP in spondyloarthritis patients with TNF-α blocker treatment indicate that new bone formation in AS occurs if inflammation is successfully treated and might be part of a healing process.

## Introduction

Ankylosing spondylitis (AS) is a chronic inflammatory disease with inflammation in the spine which can lead to bone erosions, new bone formation, and ankylosis in the spine. Inflammation is partly mediated by tumor necrosis factor-alpha (TNF-α) [1]. Treatment of patients with active AS with the currently approved TNF-blocking agents infliximab [2], etanercept [3], and adalimumab [4] has been shown to be highly effective for the improvement of signs, symptoms, and function and the reduction of both C-reactive protein (CRP) and active inflammation in the sacroiliac joints and the spine as shown by magnetic resonance imaging [5]. Histopathological studies from intervertebral discs [6], femoral heads [7,8], sacroiliac joint [1,9], manubriosternal junction [10], and facet joints [11,12] suggest that a subchondral inflammation at the interface between bone and cartilage – a subchondral osteitis – could be the primary site of the AS immunopathology. In recent histopathological studies, we have reported mononuclear cell aggregates, cartilage degradation, high microvessel density, and increased osteoclastic activity at sites of acute inflammation [8]. In areas of less or no inflammation, repair mechanisms such as increased osteoblast activity were seen [8]. The analysis of serum biomarkers reflecting inflammation, bone destruction, and new bone formation could be helpful for a better understanding of the sequence of events in the spine. Therefore, we analysed vasoendothelial growth factor (VEGF), metalloproteinase-3 (MMP-3), and bone-specific alkaline phosphatase (BALP) in the sera of different cohorts of patients with axial spondyloarthritis (SpA). VEGF is a potent angiogenic vasoactive molecule that increases vascular permeability and is a specific mitogen for endothelial cells [13]. It has been suggested that it correlates well with inflammation and disease activity in spondyloarthritides [14]. MMP-3 degrades extracellular matrix proteins and is involved in disruptive events in the cartilage and bone of inflamed joints [15]. Recent publications suggest that it is closely related to inflammation and high disease activity in AS [16-18] and that it also correlates significantly to radiographic progression [19]. BALP was chosen as an indicator for new bone formation because skeletal growth and bone consolidation correlate well with serum levels of bone biochemical markers like BALP and osteocalcin [20].

## Materials and methods

### Tumor necrosis factor-alpha blocker-naïve patients

In the era before TNF-α-blocking treatment, we had collected sera from AS patients at baseline and after 1 and 2 years. All 71 AS patients who were included in this study belong to the German Spondyloarthritis Inception Cohort (GESPIC) and were selected because sera at baseline, after 1 year, and after 2 years were available. The mean age of this cohort was 37.75 ± 10.67 years, the mean disease duration was 5.28 ± 2.66 years, 61.97% of the patients were male, and 81.69% were HLA-B27-positive. The Bath Ankylosing Spondylitis Disease Activity Index (BASDAI), erythrocyte sedimentation rate (ESR), and CRP were taken at all time points. Serum levels of BALP, VEGF (n = 50), and MMP-3 were measured at the given time points. All patients gave informed consent for this study. Permission for this study was given by the local ethics committee of the Charité Berlin, Campus Benjamin Franklin. The majority of patients used non-steroidal anti-inflammatory drugs, and none of them was on disease-modifying anti-rheumatic drugs or steroids.

### Adalimumab-treated patients

All 34 patients treated with adalimumab were diagnosed as axial SpA [21]. Twenty-two patients with the diagnosis of AS according to the modified New York criteria (mean age: 44.73 ± 12.09 years, mean disease duration: 15.33 ± 10.15 years) and 12 patients with non-radiographic axial SpA [22] diagnosed by magnetic resonance imaging and typical clinical symptoms (mean age: 31.67 ± 6.80 years, mean disease duration: 3.58 ± 2.91 years) were included in this analysis. These patients either were part of other studies that have been reported elsewhere [22,23] or were treated open-label. Sera from AS patients were obtained during another clinical trial [23] or during open-label therapy. The mean age of all patients treated with adalimumab was 40.12 ± 12.2 years, and the mean disease duration was 10.63 ± 9.9 years. Serum was taken before and 12 and 36 to 52 weeks after treatment with adalimumab was initiated. Each patient received 40 mg of adalimumab subcutaneously every other week. The majority of patients used non-steroidal anti-inflammatory drugs, and none of them was on disease-modifying anti-rheumatic drugs or steroids. All patients gave informed consent for this study.

### Serum enzyme-linked immunosorbent assay of VEGF, MMP-3, and BALP

Serum levels of VEGF, MMP-3, and BALP were measured by commercially available enzyme-linked immunosorbent assay (ELISA) kits: Quantikine^® ^Human MMP-3 (total) Immunoassay (R&D Systems, Wiesbaden-Nordenstadt, Germany) for MMP-3, Quantikine^® ^Human VEGF Immunoassay (R&D Systems) for VEGF, and MetraBAP (Quidel, San Diego, USA) for BALP). Analysis was performed in accordance with the instructions of the manufacturers. For reasons of availability, 21 of the 71 TNF-α-naïve AS patients were excluded from VEGF analysis.

### Statistical analysis

Serum levels of MMP-3, VEGF, and BALP between baseline and different time points were analysed using the Wilcoxon test. The correlation of differences in the serum levels of MMP-3, VEGF, BALP, and CRP before and after treatment with adalimumab was also analysed by using the Spearman rank correlation coefficient. The non-parametric Brunner test was applied to compare changes in serum levels of VEGF, MMP-3, or BALP between AS and non-radiographic axial SpA patients by taking the baseline status of VEGF, MMP-3, or BALP into account.

## Results

### Correlation of clinical parameters with serum levels of VEGF, MMP-3, and BALP in patients with spondyloarthritis

We correlated the serum levels of VEGF, MMP-3, and BALP to clinical parameters in the cohort of TNF-α blocker-naïve AS patients. VEGF correlated well to CRP (*r *= 0.307, *P *= 0.030), ESR (n = 49) (*r *= 0.370, *P *= 0.009), and BASDAI (*r *= 0.340, *P *= 0.018) at baseline and after 2 years (CRP: *r *= 0.361, *P *= 0.010; ESR: *r *= 0.372, *P *= 0.011; BASDAI: *r *= 0.496, *P *= 0.003). MMP-3 correlated well to CRP at baseline (*r *= 0.291, *P *= 0.014) but not to CRP after 2 years (*r *= 0.112, *P *> 0.05). MMP-3 did not correlate to ESR at baseline or after 2 years (*r *= 0.011, *P *> 0.05 and *r *= -0.139, *P *> 0.05) or to BASDAI (*r *= -0.039, *P *> 0.05 and *r *= -0.062, *P *> 0.05). BALP correlated to CRP at baseline (*r *= 0.333, *P *= 0.005) but not after 2 years (*r *= 0.012, *P *> 0.05). We did not find a correlation of BALP to ESR (*r *= 0.176, *P *> 0.05 and *r *= -0.082, *P *> 0.05) or BASDAI (*r *= 0.069, *P *> 0.05 and *r *= -0.222, *P *> 0.05) at baseline or after 2 years.

### No changes in serum levels of VEGF, MMP-3, and BALP in a tumor necrosis factor-alpha blocker-naïve cohort over a 2-year observation period

**Figure 1:**
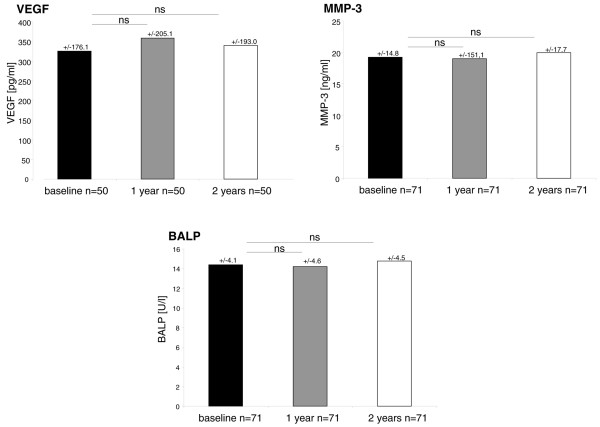
Mean serum levels of vasoendothelial growth factor (VEGF), metalloproteinase-3 (MMP-3), and bone-specific alkaline phosphatase (BALP) at baseline and after 1 and 2 years in a tumor necrosis factor-alpha blocker-naïve cohort of ankylosing spondylitis patients. ns, not significant.

The serum level of VEGF was 325.34 ± 176.09 pg/mL at baseline and did not change significantly if compared with serum levels after 1 year (358.08 ± 205.06 pg/mL, *P *> 0.05) and 2 years (339.14 ± 198.03 pg/mL, *P *< 0.05) (Figure 1). Similarly, serum levels of MMP-3 remained stable and were 19.23 ± 14.81 ng/mL at baseline and 19.03 ± 15.12 ng/mL (*P *> 0.05) after 1 year and 19.96 ± 17.70 ng/mL after 2 years (*P *> 0.05) (Figure 1). Also, the serum level of BALP did not show any significant change over the course of 2 years: 14.37 ± 4.08 U/L after baseline, 14.18 ± 4.56 U/L after 1 year (*P *> 0.05), and 14.75 ± 4.45 U/L after 2 years (*P *> 0.05) (Figure 1) on the group level.

### Serum levels of VEGF, MMP-3, and BALP during tumor necrosis factor-alpha blocker treatment with adalimumab

**Figure 2:**
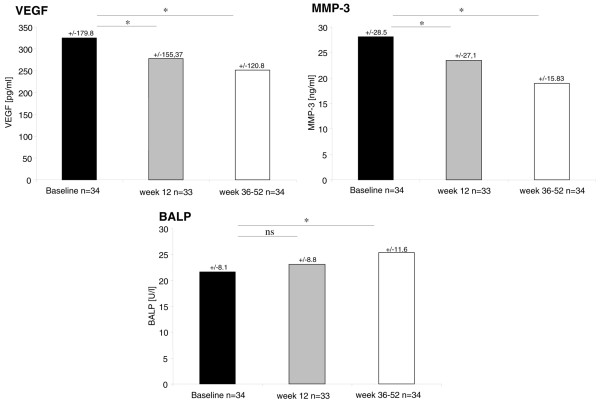
Mean serum levels of vasoendothelial growth factor (VEGF), metalloproteinase-3 (MMP-3), and bone-specific alkaline phosphatase (BALP) in patients with axial spondyloarthritis before and after 12 and 36 to 52 weeks of treatment with adalimumab. *P < 0.05. ns, not significant.

The majority of patients with axial SpA reported a major benefit from TNF-α therapy with adalimumab. The mean BASDAI decreased by more than 3 points after 36 to 52 weeks (*P *< 0.001). ESR (*P *= 0.007) and CRP (*P *< 0.001) also decreased significantly. Accordingly, a decrease of VEGF and MMP-3 serum levels was observed: serum levels of VEGF were 324.6 ± 179.8 pg/mL before therapy and decreased significantly to 277.1 ± 155.37 pg/mL after 12 weeks and to 250.7 ± 120.8 pg/mL after 36 to 52 weeks of therapy (*P *< 0.001). Serum levels of MMP-3 were 28.0 ± 28.5 ng/mL before therapy and decreased significantly to 23.5 ± 27.1 ng/mL after 12 weeks and to 19.02 ± 15.83 ng/mL after 36 to 52 weeks of therapy (*P *= 0.022). In contrast, the serum level of BALP increased significantly during therapy: from 21.6 ± 8.1 U/L before therapy to 23.2 ± 8.8 U/L after 12 weeks and to 25.4 ± 11.6 U/L after 36 to 52 weeks of therapy (*P *< 0.001) (Figure 2).

### Differences in serum levels of VEGF, MMP-3, and BALP in response to adalimumab in ankylosing spondylitis and non-radiographic axial spondyloarthritis

We also addressed the question of whether serum levels of VEGF, MMP-3, and BALP differed significantly between patients with non-radiographic axial SpA (n = 12) and AS (n = 22) diagnosed by the modified New York criteria. Serum levels of VEGF decreased significantly in patients with AS (308.90 ± 175.74 pg/ml at baseline and 224.10 ± 108.90 pg/ml after 36 to 52 weeks, *P *= 0.004) and non-significantly in patients with early axial SpA (353.40 ± 191.26 pg/ml at baseline and 299.56 ± 130.87 pg/ml after 36 to 52 weeks, *P *= 0.084). Serum levels of MMP-3 also decreased significantly in AS (30.12 ± 32.16 ng/ml at baseline and 20.31 ± 18.77 ng/ml after 36 to 52 weeks, *P *= 0.022) and non-significantly in early axial SpA (24.20 ± 21.07 ng/ml at baseline and 16.63 ± 8.34 ng/ml after 36 to 52 weeks of therapy, *P *= 0.388). Interestingly, serum levels of BALP increased significantly in AS (21.43 ± 9.21 U/l at baseline and 26.50 ± 13.17 U/l after 36 to 52 weeks of therapy, *P *= 0.001) but less clearly and non-significantly in non-radiographic axial SpA (22.01 ± 5.90 U/l at baseline and 23.46 ± 7.96 U/l after 36 to 52 weeks of therapy, *P *= 0.308). Comparisons of the change in scores between AS and non-radiographic axial SpA adjusted for the baseline status revealed no significant differences (VEGF: *P *= 0.294, MMP-3: *P *= 0.324, BALP: *P *= 0.128).

### Correlation between differences in serum levels of VEGF, MMP-3, BALP, and C-reactive protein before and after 36 to 52 weeks of treatment with adalimumab

**Figure 3:**
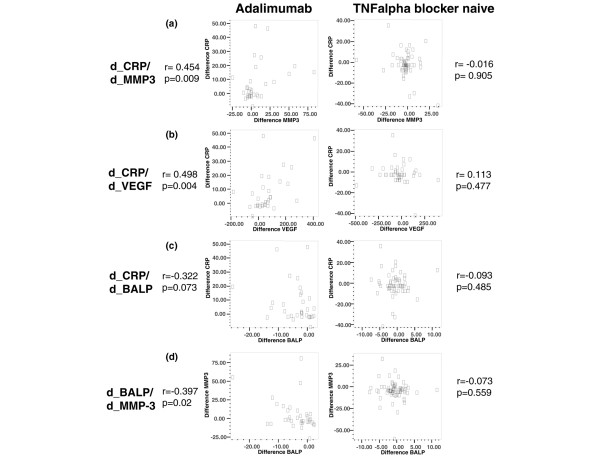
Correlation of differences in serum levels at baseline and 1 year of **(a) **metalloproteinase-3 (d_MMP-3) and C-reactive protein (d_CRP), **(b) **vasoendothelial growth factor (d_VEGF) and d_CRP, **(c) **bone-specific alkaline phosphatase (d_BALP) and d_CRP, and **(d) **d_MMP-3 and d_BALP.

Next, we correlated the differences of biomarker serum levels at baseline and after 36 to 52 weeks of treatment with adalimumab (Figure 3). Interestingly, the difference of serum levels of VEGF between weeks 36 to 52 and baseline (d_VEGF) and d_MMP-3 correlated nicely with d_CRP (VEGF: *r *= 0.498, *P *= 0.004; MMP-3: *r *= 0.454, *P *= 0.009) and we observed a non-significant tendency of negative correlation between d_BALP and d_CRP (*r *= -0.322, *P *= 0.073). Most interestingly, if d_BALP and d_MMP-3 were analysed, a statistically significant negative correlation was found (*r *= -0.398, *P *= 0.02). A correlation analysis of d_BALP and d_VEGF also revealed a tendency of negative correlation (*r *= -0.214, *P *> 0.05). Such correlations of differences in d_BALP and d_MMP-3 over time were not observed in the sera of AS patients from the TNF-α blocker-naïve cohort.

## Discussion

In this study, we measured the serum levels of VEGF, MMP-3, and BALP in a cohort of TNF-α blocker-naïve AS patients during an observation period of 2 years and compared these results with a cohort of adalimumab-treated AS patients. If we correlated serum biomarkers to clinical parameters in TNF-α blocker-naïve AS patients, we observed a good correlation of VEGF with CRP, ESR, and BASDAI. This is in line with previous reports showing that VEGF is a good indicator of high disease activity in patients with AS [14,24,25]. VEGF serum levels were higher in AS patients than in healthy controls and showed a significant correlation with BASDAI, ESR, and CRP in a previous study [24]. We could also observe a correlation of MMP-3 with CRP at baseline. However, the correlation of MMP-3 with ESR and BASDAI was not significant. Similar results were reported recently by Woo and colleagues [26]. Nonetheless, other studies revealed significant correlations of serum levels of MMP-3 with CRP, ESR, and BASDAI (respectively) [17] or with ESR and BASDAI but not with CRP [27]. We obtained no clear correlation of serum levels of BALP with parameters of disease activity. Besides a correlation of BALP with CRP at baseline, all other analysis revealed no significant correlations. Such a correlation of BALP with CRP was also observed in a study of 56 AS patients [28] but not in a recent analysis of 26 AS patients [26]. Thus, current data, including our present study, give evidence that serum levels of VEGF are a good parameter to indicate disease activity in AS while there are some contradictory results about the use of MMP-3 serum levels to indicate disease activity. Finally, BALP seems not to be a sensitive parameter to indicate higher disease activity in AS.

We next addressed the question of whether serum levels of these biomarkers change in the same patients during an observation period of 2 years without treatment with a TNF-α blocker. Interestingly, serum levels of all three biomarkers in this cohort remained stable during this period without significant changes in comparison with baseline, which is reported for the first time in the case of MMP-3 serum levels. A longitudinal study of VEGF and BALP serum levels during an observation period of 2 years was not reported before, but stable serum levels of VEGF and BALP were also seen in a cohort of 78 AS patients over the course of 24 weeks [24,25]. However, in the cohort of SpA patients with the TNF blocker adalimumab, we observed a significant decrease of MMP-3 after 36 to 52 weeks. The difference of serum levels of MMP-3 after 36 to 52 weeks compared with baseline correlated significantly to the decrease of CRP. A decrease of MMP-3 levels during treatment with TNF-α blocker treatment was also reported recently in AS patients receiving infliximab or etanercept [18,26,29].

Adalimumab-treated SpA patients also displayed a significant decrease of VEGF after 36 to 52 weeks. The difference of serum levels of VEGF after 36 to 52 weeks compared with baseline correlated significantly to a decrease of CRP (difference from 36 to 52 weeks to baseline). The influence of TNF-α blocker agents on serum levels of VEGF has been reported only for infliximab-treated patients. In 201 infliximab-treated AS patients, serum levels of VEGF decreased significantly during treatment within an observation period of 24 weeks [24]. Our data indeed support the view that a decrease of VEGF serum levels is a good indicator of response in TNF-α blocker-treated AS patients.

In our cohort of adalimumab-treated SpA patients, the serum levels of BALP increased significantly during 36 to 52 weeks of treatment. This is in line with recent reports of AS patients treated with infliximab [25] and etanercept [26]: in 26 AS patients treated with etanercept, BALP serum levels increased significantly during an observation period of 12 weeks; this correlated to osteocalcin serum levels, which is another marker for increased osteoblast activity [26]. Seventy-eight infliximab-treated AS patients showed a significant increase of BALP serum levels after 102 weeks but not after 24 weeks of observation [25]. Interestingly, the differences of serum levels of BALP and MMP-3 between baseline and 36 to 52 weeks displayed a significant negative correlation in our analysis. This finding supports the idea that successful treatment of inflammation is closely linked to the induction of new bone formation [30]. MMP-3 was recently suggested as an independent predictor of new bone formation [19]. Such a high level of MMP-3 at baseline is suggestive of structural damage that might undergo repair if inflammation is successfully treated [30].

Other studies analysing biomarkers during treatment with TNF-α-blocking agents also observed a switch from degradation to anabolic mechanisms. During treatment of AS patients with etanercept, cartilage degradation illustrated by serum levels of type II collagen fragment (C2C) decreased during treatment whereas the serum level of procollagen type I N-terminal propeptide (PINP), a marker for new bone formation, increased significantly [31]. Increased serum levels of BALP might reflect not only new bone formation in repair tissue of inflammatory lesions in AS but also a more general increase of bone mineral density in AS patients treated with TNF-α-blocking agents [32].

The modified New York criteria normally have to be fulfilled for a diagnosis of AS. This implies the presence of sacroiliitis as shown by x-rays [33]. Our cohort of axial SpA patients treated with TNF-α blockers included patients with AS and non-radiographic axial SpA [21,22]. Subdividing these two groups (AS n = 22 and non-radiographic AS n = 12) and performing a subanalysis revealed the interesting result that serum levels of VEGF, MMP-3, and BALP did not differ significantly in the two groups of patients. Moreover, serum levels of MMP-3 and VEGF both decreased during therapy and BALP increased in both groups of patients. However, this was significant in AS patients only.

## Conclusions

Biomarkers in the serum of patients have been increasingly used to get a better idea about bone-destructive and bone-anabolic phases in different forms of arthritides, including spondyloarthritides. For the first time, here we report a negative correlation between bone-degrading parameters and parameters of new bone formation during treatment with a TNF blocker such as adalimumab in axial SpA.

## Abbreviations

AS: ankylosing spondylitis; BALP: bone-specific alkaline phosphatase; BASDAI: bath ankylosing spondylitis disease activity index; CRP: C-reactive protein; ELISA: enzyme-linked immunosorbent assay; ESR: erythrocyte sedimentation rate; MMP-3: metalloproteinase-3; SpA: spondyloarthritis; TNF-α: tumor necrosis factor-alpha; VEGF: vasoendothelial growth factor.

## Competing interests

HA has received speaking fees from Abbott Laboratories (Abbott Park, IL, USA), Schering-Plough Corporation (Kenilworth, NJ, USA), and Centocor, Inc. (Horsham, PA, USA) (less than USD $10,000 each). MR has received speaking fees from Abbott Laboratories, Schering-Plough Corporation, Centocor, Inc., Wyeth (Madison, NJ, USA), and Novartis International AG (Basel, Switzerland) (less than USD $10,000 each). JS has received speaking fees, consulting fees, and/or honoraria from Schering-Plough Corporation, Wyeth, Abbott Laboratories, Roche (Basel, Switzerland), and Pfizer Inc (New York, NY, USA) (less than USD $10,000 each). The other authors declare that they have no competing interests.

## Authors' contributions

HA developed the study, analysed the data, and drafted the manuscript. LJ participated in the data collection, performed the data analysis, and helped in the drafting of the manuscript. JL performed the statistical analysis and helped in the drafting of the manuscript. RH participated in the data collection and the data analysis. MR participated in the development of the study and the drafting of the manuscript. JS conceived the study and drafted the manuscript. All authors have read and approved the final manuscript.
